# A novel Rho-dependent pathway that drives interaction of fascin-1 with p-Lin-11/Isl-1/Mec-3 kinase (LIMK) 1/2 to promote fascin-1/actin binding and filopodia stability

**DOI:** 10.1186/1741-7007-10-72

**Published:** 2012-08-10

**Authors:** Asier Jayo, Maddy Parsons, Josephine C Adams

**Affiliations:** 1 Randall Division of Cell and Molecular Biophysics, King's College London, Guy's Campus, London SE1 1UL, UK; 2 School of Biochemistry, University of Bristol, Bristol BS8 1TD, UK

## Abstract

**Background:**

Fascin-1 is an actin crosslinking protein that is important for the assembly of cell protrusions in neurons, skeletal and smooth muscle, fibroblasts, and dendritic cells. Although absent from most normal adult epithelia, fascin-1 is upregulated in many human carcinomas, and is associated with poor prognosis because of its promotion of carcinoma cell migration, invasion, and metastasis. Rac and Cdc42 small guanine triphosphatases have been identified as upstream regulators of the association of fascin-1 with actin, but the possible role of Rho has remained obscure. Additionally, experiments have been hampered by the inability to measure the fascin-1/actin interaction directly in intact cells. We investigated the hypothesis that fascin-1 is a functional target of Rho in normal and carcinoma cells, using experimental approaches that included a novel fluorescence resonance energy transfer (FRET)/fluorescence lifetime imaging (FLIM) method to measure the interaction of fascin-1 with actin.

**Results:**

Rho activity modulates the interaction of fascin-1 with actin, as detected by a novel FRET method, in skeletal myoblasts and human colon carcinoma cells. Mechanistically, Rho regulation depends on Rho kinase activity, is independent of the status of myosin II activity, and is not mediated by promotion of the fascin/PKC complex. The p-Lin-11/Isl-1/Mec-3 kinases (LIMK), LIMK1 and LIMK2, act downstream of Rho kinases as novel binding partners of fascin-1, and this complex regulates the stability of filopodia.

**Conclusions:**

We have identified a novel activity of Rho in promoting a complex between fascin-1 and LIMK1/2 that modulates the interaction of fascin-1 with actin. These data provide new mechanistic insight into the intracellular coordination of contractile and protrusive actin-based structures. During the course of the study, we developed a novel FRET method for analysis of the fascin-1/actin interaction, with potential general applicability for analyzing the activities of actin-binding proteins in intact cells.

## Background

Cell protrusions are dynamic and morphologically varied extensions of the plasma membrane, supported by the actin cytoskeleton, that are essential for cell migration. Fascin-1 is a prominent actin-bundling protein that characterizes the filopodia, microspikes, and dendrites of mesenchymal, neuronal, and dendritic cells, respectively, and also contributes to filopodia, podosomes, and invadopodia in migratory vascular smooth muscle cells and cancer cells [1-4]. Fascin-1 is absent from most normal adult epithelia, yet is upregulated in human carcinomas arising from a number of tissues. There is evidence that fascin-1 supports the migratory and metastatic capacities of carcinomas [3-7]. Fascin-1 is an independent indicator of poor prognosis in non-small-cell lung carcinomas and colorectal, breast, and other carcinomas [4,8-11]. In colon, breast, or prostate carcinomas, fascin-1 protein correlates with increased frequency of metastasis [7,10,11]. Fascin-1 is thought to be the target of macroketone, which is under investigation as an anti-cancer agent [10]. For these reasons, identification of the signaling pathways that regulate fascin-1 in carcinoma cells has become an important focus of research.

Actin bundling has been shown *in vitro *to be a conserved activity of fascins [12-15]. In filopodia, fascin-1 molecules crosslink actin filaments into parallel bundles, yet also move dynamically in and out of the bundle, which may allow for bundle turning and bending [16]. F-actin cross linking by fascin-1 involves the N-terminal and C- terminal domains of fascin-1, and a major mechanism that inhibits the actin-bundling activity of fascin-1 is the phosphorylation of an N-terminal motif (S39 in human fascin-1) by conventional isoforms of protein kinase C (cPKC) [17-19]. cPKC phosphorylation of S39 inhibits actin binding and drives the formation of a complex between phosphorylated fascin-1 and active cPKC, resulting in a diffuse cytoplasmic distribution of fascin-1 [18,20]. In migrating carcinoma cells, fascin-1 and cPKC associate dynamically in filopodia and at cell edges, and the cycling of phosphorylated fascin-1 is necessary for directional cell migration and experimental metastasis [5,19]. Rac1 is a major upstream regulator of both these activities of fascin-1; it promotes the bundling of F-actin by fascin-1 in lamellipodia [21], and drives the formation of a complex between phosphorylated fascin-1 and active cPKC, through a pathway involving group I p21-activated kinases [19].

Effective cell migration depends on integration of the F-actin cytoskeleton of protrusions with the contractile actomyosin stress fibers in the cell body [22]. The molecular basis of this integration is not well understood, but fascin-1 is known to associate with stress fibers under conditions associated with moderate extracellular matrix (ECM) adhesion, such as on mixed thrombospondin-1/fibronectin (FN) surfaces or under conditions of partial impairment of cell spreading on FN caused by a function-pertubing antibody to α5 integrin [20,23,24]. In fish keratocyes, fascin-containing filopodia contribute actin filament bundles into myosin II-containing stress fibers or fold back to incorporate into lamellipodial F-actin arcs [25]. The small guanine triphosphatase (GTPase) Rho is a major regulator of cell contractility that acts antagonistically to Rac in several cellular pathways [26]. but whether Rho regulates fascin-1 is unknown. Several lines of evidence indicate functional links between fascin-1 protrusions and the contractile focal adhesions that are promoted by active Rho; the phosphofascin-1/cPKC complex regulates the balance between protrusions and focal adhesions in mesenchymal cells, and depletion of fascin-1 from colon carcinoma cells inhibits focal adhesion disassembly and prevents filopodia formation [5,18]. Whether Rho participates in these processes is unknown. Although overexpression of constitutively active Rho alters fascin-1 localization in quiescent fibroblasts, dominant-negative Rho does not inhibit the long-lived fascin-1 protrusions of cells adherent on thrombospondin-1 [21]. Tenascin-C, another ECM glycoprotein that activates assembly of fascin-1 protrusions, suppresses Rho activity in fibroblasts by a syndecan-4 dependent pathway [27-30].

In this study, we investigated the hypothesis that fascin-1 is a functional target of Rho and identified a pathway from Rho via Rho kinases to p-Lin-11/Isl-1/Mec-3 kinases (LIMK)1 and LIMK2. We found that LIMK1/2 is a novel positive regulator of the fascin-1/actin interaction and is a novel interaction partner of fascin-1. These data have important implications for consideration of the role of fascin-1 in carcinoma metastasis.

## Results

### RhoA supports the interaction of fascin-1 with actin in migrating cells

**Figure 1:**
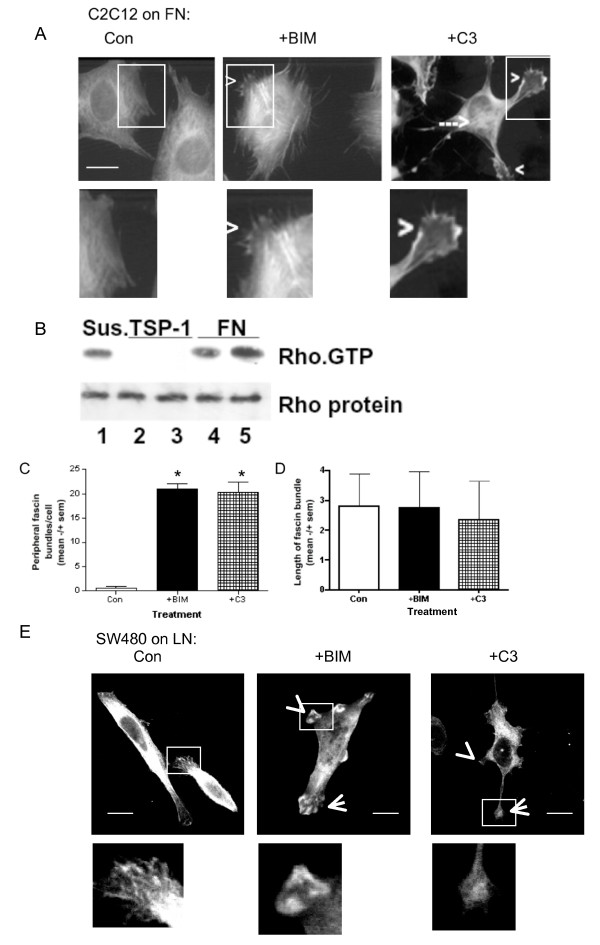
**Rho inhibition modulates peripheral fascin-containing protrusions**. **(A) **C2C12 cells (control or treated with the indicated pharmacological inhibitors), were plated onto 50 nmol/l fibronectin (FN) for 1 hour, then fixed and stained for fascin-1. Arrowheads indicate examples of fascin-containing protrusions, dotted arrow indicates fascin in association with stress fibers. Boxed areas are enlarged below. Scale bars, 10 μm. **(B) **Representative results of rhotekin-Rho-binding domain (RBD) pull-down of Rho-guanine triphosphate (GTP) from C2C12 cells adherent on 30 nmol/l FN or thrombospondin-1 for 1 hour, or suspended for 90 minutes over BSA-coated plastic. **(C,D) **Quantification of **(C) **numbers and **(D) **length of peripheral fascin bundles in C2C12 cells adherent for 1 hour on 50 nnmol/l FN after each treatment. Each column represents the mean from 70 to 100 cells from 3 independent experiments; bars indicate SEM. *P < 0.001 versus control. **(E) **SW480 cells (control or treated with the indicated pharmacological inhibitors), were plated onto 15 nnmol/l laminin (LN) for 2 hours, then fixed and stained for fascin-1. Arrowheads indicate examples of fascin-containing protrusions; boxed areas are enlarged below. Scale bars, 10 μm.

To investigate the novel hypothesis that Rho activity regulates fascin-1, we used two cell systems: mouse C2C12 skeletal myoblasts and human SW480 colon carcinoma cells. In both of these cell types, fascin-1-containing protrusions are known to be important for ECM-dependent cytoskeletal reorganizations and cell migration [5,20,31]. C2C12 mouse skeletal myoblasts adherent on FN undergo transient ruffling during attachment and spreading, followed by strong phosphorylation and complexing of fascin-1 with conventional PKC as focal adhesions assemble and then stabilize [14,27,31]. Thus, after 1 hour of adhesion to FN, fascin-1 has a diffuse distribution, and there are few fascin-1-positive cell protrusions (Figure 1A, Con). In C2C12 cells treated with bisindolylmaleimide I (BIM) to inhibit cPKC, fascin-1 was increased in bundles at cell edges and was also aligned with stress fibers, confirming that PKC-dependent phosphorylation antagonizes the actin-bundling capacity of fascin-1 [17-20] (Figure 1A).

FN-adherent C2C12 cells have significant levels of endogenous active Rho-guanine triphosphate (GTP) relative to cells adherent to thrombospondin-1 (Figure 1B). Under conditions of Rho inhibition by C3 exotoxin, C2C12 cells adherent to FN have irregular shapes, with increased fascin-1 bundles at cell edges (Figure 1A). These observations were confirmed by scoring the numbers of peripheral fascin-containing bundles in adherent cells. BIM or C3 treatments increased the number of bundles, but did not alter the lengths of bundles containing fascin-1 (Figure 1C,D). Increased association of fascin-1 with microfilament bundles within the cell body was also seen in many C3-treated cells (Figure 1A). The effects of BIM and C3 were confirmed in SW480 colon carcinoma cells undergoing Rac-dependent migration on laminin (LN) by mechanisms previously identified to depend functionally on fascin-1-dependent filopodia, dynamic fascin/PKC complexing, and focal adhesion turnover [5,15]. BIM treatment of SW480 cells on LN resulted in more irregular morphologies with non-polarized formation of fascin-1-positive protrusions at cell margins (Figure 1E). SW480 typically contain relatively few stress fibers, and the effects of C3 on fascin-1 relocalization to cell edges was less pronounced in these cells (Figure 1E, insets). Rho activity in migrating SW480 cells and its effective inhibition by C3 exotoxin was confirmed by measurement of RhoA activity under the different experimental conditions (see Additional file 1, Figure S1A). Together, these data implicate Rho activity in regulation of the dynamic balance of fascin-1 interactions with F-actin.

**Figure 2:**
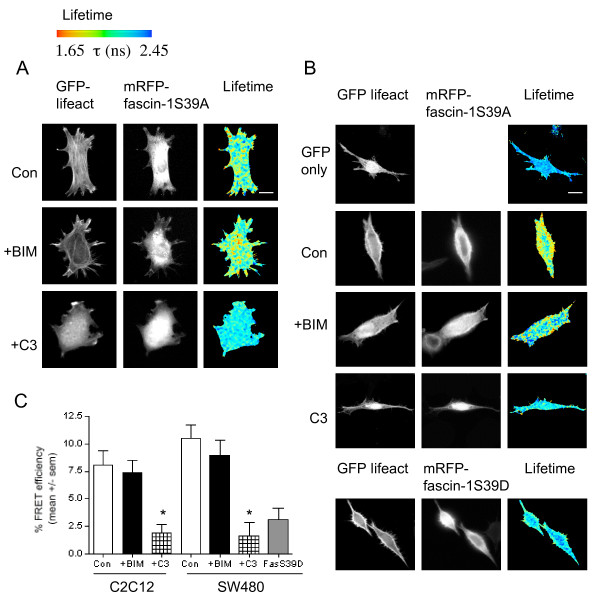
**Rho activity promotes the interaction of fascin-1 with actin: detection by a novel fascin-1/lifeact fluorescence resonance energy transfer (FRET) system**. **(A,B) **Measurement of the interaction of monomeric red fluorescent protein (mRFP)-fascin-1S39A with green fluorescent protein (GFP)-lifeact in **(A) **C2C12 cells on fibronectin (FN) or **(B) **SW480 cells on laminin (LN). Cells transiently transfected with the indicated plasmids were plated on **(A) **FN for 1 hour, or **(B) **LN for 2 hours, without or with pre-treatment with the indicated inhibitors, then fixed, mounted, and imaged using fluorescence lifetime imaging microscopy (FLIM) to measure FRET. In each panel, intensity multiphoton GFP (donor) images are shown with the corresponding epifluorescence image for mRFP (acceptor). **(B) **Representative images of GFP and lifetime plot in the absence of an acceptor, or in presence of mRFP-fascin-1S39D, which does not bundle F-actin. In each panel, lifetime images are presented in a blue-to-red pseudocolor scale with red as short lifetime. **(C)**,Percentage FRET efficiency under each experimental condition. Each column represents the mean from eight to twelve cells per condition and three independent experiments; bars indicate SEM. *P < 0.001 versus control.

To obtain precise evidence that Rho activity can regulate fascin-1, we tested the effect of Rho inhibition on the interaction of fascin-1 with actin, using fluorescence lifetime imaging microscopy (FLIM) to measure fluorescence resonance energy transfer (FRET). The abundance of actin in cells, coupled with issues of the conformational availability of fluorophores, has so far hindered attempts to measure interactions between fluorescently-tagged actin and its binding partners by FRET/FLIM. Thus, to measure the fascin-1/actin interaction directly, we took a novel approach, using green fluorescent protein (GFP)-tagged lifeact as the FRET donor. Lifeact is a peptide of 17 amino acids, which is derived from yeast, and binds specifically and reversibly to F-actin in live cells without interfering with actin dynamics [32]. To set up conditions to measure the fascin-1/actin interaction without modulation or interference by dynamic fascin-1 phosphorylation, we used as the FRET acceptor in these experiments a fascin-1 mutant, fascin-1S39A, which binds actin but does not interact with cPKC [18,19], The monomeric red fluorescent protein (mRFP)-tagged fascin-1S39A showed strong FRET with GFP-lifeact in both FN-adherent C2C12 cells and SW480 cells on LN (Figure 2A,B (Con cells); Figure 2C shows quantification from multiple cells). FRET efficiency between GFP-lifeact and mRFP-fascin1S39A was strong at the cell peripheries and was also often detected in cell bodies (Figure 2A.B; Con cells). The interaction of phosphomimetic mRFP-fascin-1S39D was minimal, with the GFP fluorescence lifetime comparable with that of cells expressing GFP-lifeact alone (shown for SW480 cells: Figure 2B,C). To confirm that the GFP-lifeact results were an accurate reflection of the distribution of F-actin in cells, cells co-expressing GFP-lifeact and mRFP-fascin1S39A were co-stained with phalloidin to visualize total F-actin, and then analyzed by FLIM. Analysis of the cell edges showed that the highest GFP-lifeact signals were found within areas with the highest intensity of phalloidin staining, thus corresponding to concentrations of F-actin (see Additional file 1, Figure S1B,C), and mRFP-fascin-1 was similarly distributed (see Additional file 1, Figure S1C). The areas of highest FRET efficiency occurred within the areas of highest intensity phalloidin staining, and overlapped partially with the concentrations of GFP-lifeact (see Additional file 1, Figure S1C). Thus, the FRET/FLIM interaction accurately reflects the portion of total F-actin that is involved in fascin-1 binding.

As expected from the initial experiments (Figure 1), treatment with BIM or C3 resulted in altered cell morphologies (Figure 2A,B). C3-treated C2C12 cells typically showed reduction of actin stress fibers within cell bodies (Figure 2A). BIM treatment did not prevent the fascin-1S39A/lifeact FRET/FLIM interaction, confirming the independence of this interaction from cPKC activity (Figure 2). In both cell types, the interaction between GFP-lifeact and mRFP-fascin-1S39A was strongly dependent on Rho activity (Figure 2A-C (C shows quantification from multiple cells)). These FRET data confirm that the direct interaction of fascin-1 with actin can be imaged using GFP-lifeact as a probe for F-actin, and that the interaction occurs preferentially with non-phosphorylated fascin-1 in intact cells. They also reveal that Rho acts in intact, ECM-adherent cells to promote the interaction of fascin-1 with actin.

### Rho inhibition does not alter levels of the fascin-1/cPKC complex

**Figure 3:**
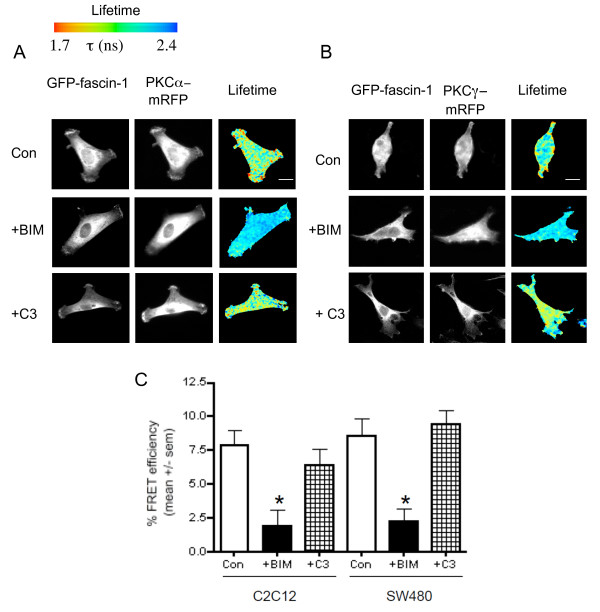
**Rho activity does not modulate the interaction of fascin-1 with conventional protein kinase C (cPKC)**. **(A) **Measurement of the interaction of green fluorescent protein (GFP)-fascin-1 with PKCα- monomeric red fluorescent protein (mRFP) in C2C12 cells on fibronectin (FN). **(B) **Measurement of the interaction of green fluorescent protein (GFP)-fascin-1 with PKCγ-mRFP in SW480 cells on laminin (LN). Cells transiently transfected with the indicated plasmids were plated on **(A) **FN for 1 hour, or **(B) **LN for 2 hours, without or with pre-treatment with the indicated inhibitors, then fixed, mounted, and imaged using fluorescence lifetime imaging microscopy (FLIM) to measure fluorescence resonance energy transfer (FRET). In each panel, intensity multiphoton GFP (donor) images are shown with the corresponding epifluorescence image for mRFP (acceptor). Lifetime images are presented in a blue-to-red pseudocolor scale with red as short lifetime. **(C)**, Percentage FRET efficiency under each experimental condition. Each column represents the mean from eight to twelve cells per condition and three independent experiments; bars indicate SEM.*P < 0.01 versus control.

To establish whether the mechanism by which Rho promotes the fascin-1/actin interaction affects the fascin-1/cPKC complex, which is a known negative regulator of actin-bundling by fascin-1, cell protrusions, and cell migration [5,18,19], we carried out FRET/FLIM measurements for the interaction of GFP-fascin-1 with cPKC-mRFP in control or inhibitor-treated cells. Both C2C12 and SW480 contain cPKC; PKCα predominates in C2C12 cells and PKCγ in SW480 cells [19,20]. When activated, both isoforms interact with phospho-fascin-1 [18,19]. In both cell types, the fascin-1/cPKC interaction was abolished in BIM-treated cells, confirming that this interaction depends on catalytically active cPKC (Figure 3A-C (C shows quantification from multiple cells)) [19]. However, C3 treatment did not alter the FRET efficiency significantly from that of control cells (Figure 3A-C). Strong decreases in GFP fluorescence lifetime, indicative of high FRET efficiency, remained detectable at the cell edges and in cell bodies (Figure 3A,B). Thus, under native cell conditions, Rho activity promotes the fascin-1/actin interaction (Figure 2), but is neutral for the fascin-1/cPKC interaction that is a known antagonist of F-actin bundling by fascin-1. These data suggest that the Rho-dependent pathway involves a novel form of regulation of fascin-1.

### Modulation of the fascin-1/actin interaction by Rho depends on Rho kinases but not on myosin-based contractility

**Figure 4:**
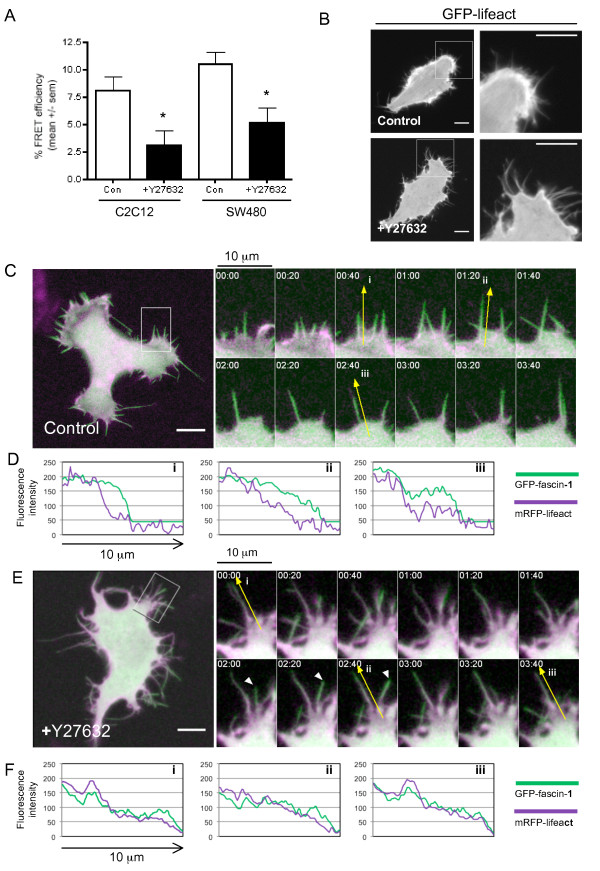
**Rho kinase activity promotes the interaction of fascin-1 with actin**. **(A) **Percentage FRET efficiency of the interaction of monomeric red fluorescent protein (mRFP)-fascin-1S39A with green fluorescent protein (GFP)-lifeact in SW480 cells on laminin (LN) under control conditions or after inhibition of Rho kinases by Y27632. Each column represents the mean from eight to twelve cells per condition and three independent experiments; bars indicate SEM. *P < 0.05 versus control. **(B) **Confocal images of the same non-fixed SW480 cell transiently expressing GFP-lifeact, before and after treatment with Y27632 (see Additional file 4, movie 1). The boxed 25 × 25 μm regions in the lefthand panels are enlarged in the zoomed right panels. Scale bars, 10 μm. **(C,E) **Fascin-1 and F-actin dynamics in SW480 cells transiently expressing GFP-fascin-1 and mRFP-lifeact, **(C) **without or **(E) **with Y27632 treatment (see Additional files 5 and 6, movies 2 and 3). **(C,E) **Left panels show representative cells from four independent confocal time-lapse movies. Scale bars, 10 μm. Right panels show zoomed images from the boxed 10 × 15 μm regions in the lefthand panels. **(D,F) **Fluorescence line-scan analysis of GFP-fascin-1 and mRFP-lifeact in a single filopodium from **(D) **control, or **(E) **Y27632-treated cells at three timepoints (i to iii). **(C,E) **Yellow arrows indicate the filopodia analyzed; **(E) **arrowheads indicate another example of an unstable filopodium. Scale bars, 10 μm.

**Figure 5:**
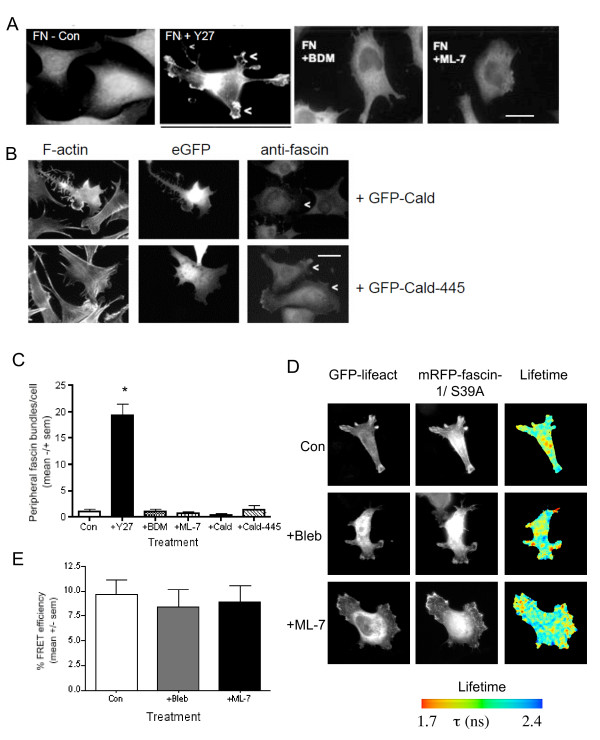
**Rho kinase activity promotes peripheral fascin-containing protrusions via a myosin-independent process**. **(A) **Confocal images of C2C12 cells after 1 hour of adhesion to 50 nmol/l fibronectin (FN), either untreated or pretreated with specified inhibitors, fixed and stained for fascin-1. Arrowheads indicate examples of peripheral fascin-actin bundles in Y27632-treated cells. Scale bars, 10 μm. **(B) **Confocal images of C2C12 cells transiently expressing green fluorescent protein (GFP)-caldesmon or an inactive GFP-caldesmon-445 mutant after 1 hour of adhesion to 50 nmol/l FN. Cells were fixed and stained either for F-actin (left panels) or fascin-1 (right panels). In the anti-fascin-1 stained samples, arrowheads indicate the transfected cells. Scale bars, 10 μm. **(C) **Quantification of peripheral fascin-1 bundles/cell under the conditions shown in **(A) **and **(B**). Data are from 75 to 125 cells/condition and 3 independent experiments. *P < 0.001 versus control. **(D) **Measurement of the interaction of mRFP-fascin-1S39A with GFP-lifeact in SW480 cells on laminin (LN). Cells transiently transfected with the indicated plasmids were plated on LN for 2 hours, without or with pre-treatment with the indicated inhibitors, then fixed, mounted, and imaged using fluorescence lifetime imaging microscopy (FLIM) to measure fluorescence resonance energy transfer (FRET). In each panel, Intensity multiphoton GFP (donor) images are shown with the corresponding epifluorescence image for mRFP (acceptor). Lifetime images are presented in a blue-to-red pseudocolor scale with red as short lifetime. **(E) **Percentage FRET efficiency under each experimental condition. Each column represents the mean from fourteen cells per condition and three independent experiments; bars indicate SEM.

To identify molecular components downstream of Rho in this novel pathway, we tested the effect of inhibiting Rho effectors that are known mediators of actin-based cell contractility. C2C12 and SW480 cells each express both isoforms of Rho-associated coiled-coil-forming kinases (Rho kinases I and II) (see Additional file 2, Figure S2A). Y27632 treatment, which inhibits Rho kinases, strongly inhibited the GFP-lifeact/mRFP-fascin-1S39A interaction in both cell types (Figure 4A shows quantification from multiple cells; for examples of individual cells, see Additional file 2, Figure S2B). The Y27632-treated cells resembled C3-treated cells in having irregular morphologies (see Additional file 2, Figure S2B). Confocal immunofluorescence microscopy for endogenous fascin-1 showed that Y27632-treated C2C12 cells on FN had more irregular morphologies, with fascin-containing protrusions around the cells (Figure 5A), again resembling the morphology of C3-treated C2C12 cells (Figure 1A). Similarly, F-actin organization at cell edges (as visualized by GFP-lifeact in SW480 cells imaged by time-lapse before and after Y27632 addition) was altered from protrusive lamellipodial edges and linear filopodia in control cells to flexible filopodia around cell margins after Y27632 treatment (Figure 4B, shown for the same cell before and after Y27632 addition; also see Additional file 3, movie 1). These protrusions were confirmed to be *de novo *filopodia, not retraction fibers, because they were assembled as new protrusions and stabilized throughout the course of the time-lapse experiments (see Additional file 3, movie 1).

The effects of Y27632 treatment were analyzed further by confocal time-lapse imaging of live SW480 cells co-expressing GFP-fascin-1 and mRFP-lifeact, in order to enable clear visualization of fascin-positive filopodia. In control cells, all filopodia contained both fascin-1 and lifeact (Figure 4C; for single-channel images, see Additional file 2, Figure S2C). Individual filopodia initiated, extended, and retracted over 1 to 3 minutes (Figure 4C, arrows; also see Additional file 4, movie 2). Line-scan analysis of fluorescence intensities for GFP-fascin-1 and mRFP-lifeact along the length of individual filopodia showed a strong fascin-1 signal along the entire length of the shaft of each filopodium, and a progressive reduction in the lifeact signal towards the tip (Figure 4D). The filopodia of Y27632-treated cells were less linear, remained extended over a longer timescale (Figure 4E shows filopodium at timepoints i to iii (arrow); see Additional file 5, movie 3), and had reduced fascin-1 intensity along the length of each filopodium (Figure 4E; see Additional file 2, Figure S2C for single-channel images). Thus, the Y27632-induced bending and altered dynamics of filopodia are probably due to alterations in organization of the core actin bundle of each filopodium and to the expected alteration in cell-body contractility caused by reductions in contractile stress fibers.

Another major mediator of cell contraction is myosin light chain kinase, (MLCK) [33]. To establish whether either MLCK or myosin activity act to inhibit actin bundling by fascin-1, FN-adherent C2C12 cells were treated with ML-7 as an inhibitor of MLCK, or 2,3-butanedione monoxime (BDM) as a broad-spectrum inhibitor of actomyosin, which has also been reported to act as a chemical phosphatase [34]. In contrast to the Y27632 treatment, no enhancement of endogenous fascin-1 in peripheral bundles was detected, indicating that the inhibitory activity of Rho kinases is not mediated by myosin-based contractility (Figure 5A; Figure 5C shows quantification of multiple cells). Similarly, expression of a dominant-negative truncated caldesmon that blocks stress-fiber assembly [35] did not promote peripheral fascin-1/actin bundles (Figure 5B; Figure 5C shows quantification of multiple cells). The possible roles of MLCK and myosin ATPase were also examined by FRET/FLIM analysis of SW480 cells co-expressing GFP-lifeact and mRFP-fascin-1S39A. Neither ML-7 nor blebbistatin (the latter tested as a specific inhibitor of myosin II ATPase) inhibited the interaction between fascin-1 and actin (Figure 5D; Figure 5E shows quantification of multiple cells). Thus, under native conditions, Rho activity promotes the interaction of fascin-1 with actin through a Rho kinase-dependent, myosin II-independent mechanism.

### Fascin-1/actin binding is promoted by interaction of fascin-1 with LIM kinases

Having identified from the above experiments that a Rho/Rho kinase/fascin-1 pathway is active in two distinct cell types, our further experiments focused on SW480 cells migrating on LN, for which signaling regulation of fascin-1 has been studied extensively [5,19]. Because the activity of Rho kinases on fascin-1 is not mediated by myosin-based contractility, we first investigated if fascin-1 might interact with a Rho kinase. SW480 express Rho kinases I and II (see Additional file 2, Figure S2A). However, using FLIM analysis, there was no FRET seen between mRFP-fascin-1S39A or mRFP-fascin-1S39D with either GFP-Rho kinase I or GFP-Rho kinase II. Furthermore, neither Rho kinase I nor Rho kinase II co-immunoprecipitated with either endogenous or overexpressed fascin-1 in SW480 cells, or with purified hexahistidine-tagged fascin-1, and fascin-1 was not a substrate in Rho kinase assays *in vitro *(data not shown). We conclude that fascin-1 is not a direct binding partner of Rho kinase I or II.

**Figure 6:**
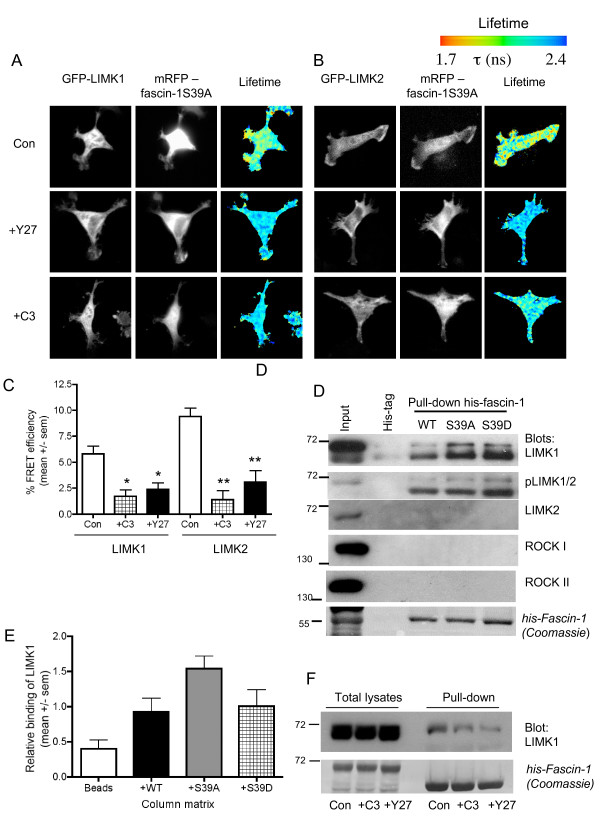
**Rho-dependent and Rho kinase-dependent interaction of fascin-1 with p-Lin-11/Isl-1/Mec-3 kinase (LIMK) (A,B)**. Measurement of the interaction of **(A) **green fluorescent protein (GFP)-LIMK1 (A), or **(B) **GFP-LIMK2, with monomeric red fluorescent protein (mRFP)-fascin-1S39A in SW480 cells on laminin (LN). Cells transiently transfected with the indicated plasmids were plated on LN for 2 hours, without or with pre-treatment with the indicated inhibitors, then fixed, mounted, and imaged using fluorescence lifetime imaging microscopy (FLIM) to measure fluorescence resonance energy transfer (FRET). In each panel, intensity multiphoton GFP (donor) images are shown with the corresponding epifluorescence image for monomeric red fluorescent protein (mRFP) (acceptor). Lifetime images are presented in a blue-to-red pseudocolor scale with red as short lifetime. **(C)**, Percentage FRET efficiency under each experimental condition. Each column represents the mean from nine to sixteen cells per condition and three independent experiments; bars indicate SEM. *P < 0.01 versus control; **P < 0.005 versus control. **(D) **Representative immunoblots from pull-downs of SW480 cell lysates with hexahistidine (6His)-tagged fascin-1 (wild-type (WT), S39A or S39D) bound to nickel-agarose beads. **(E) **Quantification of LIMK1 binding to fascin-1 bead matrices. For each matrix, LIMK1 binding was ratioed to binding to the bead-only matrix, based on quantification of grayscale images in ImageJ software (http://rsb.info.nih.gov/ij/download.html). Each column represents mean values from three independent experiments; bars indicate SEM. **(F) **Demonstration that LIMK1 binding to 6His-fascin-1 depends on Rho and Rho kinase activities. Representative of three independent experiments. **(D,F) **Molecular markers are in kDa.

The LIM kinases, LIMK1 and LIMK2, are well-established substrates and effectors of Rho kinases. LIMK1/2 are dual-specificity kinases that function in organization of the actin and microtubule cytoskeletons, cell-motility processes including cancer metastasis, and cell cycle progression [36]. In migrating SW480 cells, endogenous LIMK1 and LIMK2 are located in the cytoplasm and at protrusive edges, where GFP-fascin-1 (expressed at very low levels under a truncated cytomegalovirus (CMV) promoter, 'specGFP'; see Methods) also concentrates (see Additional file 6, Figure S3A). To test for a possible direct interaction between fascin-1 and LIMK1/2, a FRET/FLIM assay was set up. In SW480 cells, robust FRET was detected between mRFP-fascin-1S39A and either GFP-LIMK1 or GFP-LIMK2 (Figure 6A-C). The interactions were also analyzed using GFP-fascin-1 as the FRET donor and mRFP-LIMK1 as the acceptor (data not shown). Interaction of mRFP-fascin-1S39A with either LIMK 1 or LIMK2 was inhibited in cells treated with either C3 or Y27632, confirming that the interaction depends on active Rho small GTPase and Rho kinases (Figure 6A-C). Rho kinases activate LIMK by phosphorylation of threonine 508 (in LIMK1) or threonine 505 (in LIMK2); this induces LIMK dimerization, autophosphorylation, and catalytic activation [36-38]. As expected, the levels of activated pT508/T505 LIMK1/2 were sharply decreased in cells treated with C3 or Y27632 (see Additional file 6, Figure S3B).

To confirm the novel fascin-1/LIMK1/2 interaction by an independent biochemical method, and to investigate whether the S39 phosphorylation site of fascin-1 has a role in the interaction, hexahistidine (6His)-tagged fascin-1 (wild-type), or 6His-fascin-1 with point mutations of S39 were expressed in *Escherichia coli*, purified with metal-affinity beads, and matched amounts loaded onto fresh metal-affinity beads. Equal protein loadings of SW480 cell lysate were passed over the beads, and bound candidate proteins were identified by immunoblotting after extensive washing. Relative to a control bead matrix, LIMK1 was found to bind to all three forms of fascin-1, indicating that S39 phosphorylation of fascin-1 does not control this interaction (Figure 6D; Figure 6F shows quantification from multiple cells). However, some enrichment of LIMK1 on fascin-1S39A matrix was detected across multiple experiments (Figure 6F). Active LIMK1/2 bound to both the wild-type and mutant fascin-1 proteins, as detected with an antibody to T508/T505-phosphorylated LIMK1/2 (Figure 6D). Very low levels of LIMK2 were detected in SW480 cells, and LIMK2 binding to 6His-fascin-1 was not detected (Figure 6D). The LIMK1 interaction with fascin-1 was specific, because binding of Rho kinase I or II was not detected (Figure 6D) in agreement with the previous FRET analyses of possible Rho kinase binding to fascin-1. Also in agreement with the FRET data, lysates from cells treated with C3 exotoxin or Y27632 showed reduced binding of LIMK1 to 6His-fascin-1 (Figure 6E). This result again indicates the importance of LIMK1 activation for its binding to fascin-1. The combined FRET and biochemical data show that Rho-dependent regulation of the fascin-1/actin interaction is achieved by activation of LIMK1/2 and an unsuspected direct interaction of fascin-1 with LIMK1/2.

### The fascin-1/LIMK1/2 interaction depends on LIMK1/2 activation and modulates filopodia dynamics

**Figure 7:**
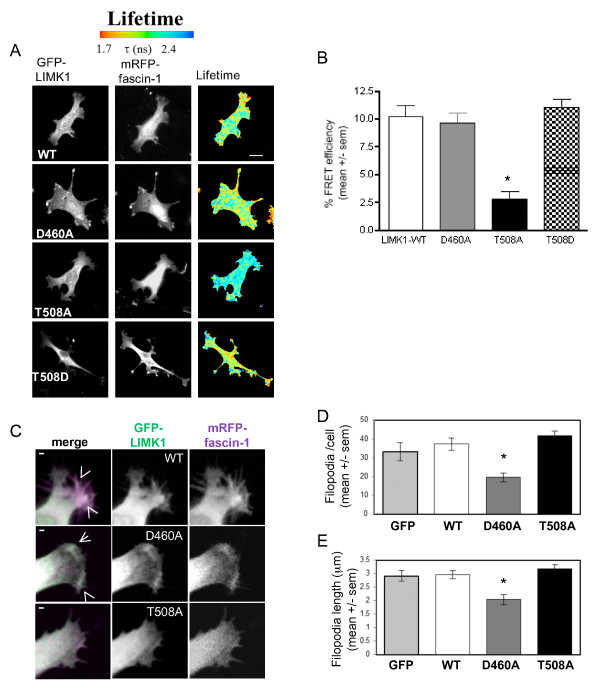
**Activation of p-Lin-11/Isl-1/Mec-3 kinase (LIMK)1 leads to its interaction with fascin-1 and affects formation of filopodia**. **(A) **Measurement of the interaction of wild-type or mutant forms of green fluorescent protein (GFP)-LIMK1 with monomeric red fluorescent protein (mRFP)-fascin-1 in SW480 cells on laminin (LN). Cells transiently transfected with the indicated plasmids were plated on LN for 2 hours, then fixed, mounted, and imaged using fluorescence lifetime imaging microscopy (FLIM) to measure fluorescence resonance energy transfer (FRET). Intensity multiphoton GFP (donor) images are shown with the corresponding epifluorescence image for mRFP (acceptor). Lifetime images are presented in a blue-to-red pseudocolor scale with red as short lifetime. **(B) **Percentage FRET efficiency under each experimental condition. Each column represents the mean from fourteen to seventeen cells per condition and three independent experiments; bars indicate SEM. *P = 0.001 versus wildtype. **(C) **Role of LIMK1 activity in organization of filopodia. Live SW480 cells transiently transfected with GFP alone or GFP-LIMK1 (wild-type (WT), D460A or T508A) and mRFP-fascin-1, and protein localizations and cell edges were imaged using confocal microscopy. Arrowheads indicate points where GFP-LIMK1 and mRFP-fascin-1 colocalize in filopodia. Scale bars, 10 μm. **(D) **The number/cell and **(E) **length of filopodia were counted from images obtained as in **(C)**, from 12 to 20 cells per condition and 4 independent experiments. *P < 0.05 versus GFP control. See Additional files 9 and 10 (movies 6 and 7) for the effects of LIMK1 mutants on filopodia.

To study the mechanism and functional role of the fascin-1/LIMK interaction in carcinoma cells, we first measured interactions of non-activatable (T508A) or catalytically inactive (D460A) forms of GFP-LIMK1 with mRFP-fascin-1 by FRET/FLIM in SW480 cells migrating on laminin. The catalytic activity of LIMK1 was not required for the interaction. By contrast, LIMK1T508A showed significantly reduced FRET with fascin-1. The phosphomimetic mutant, LIMK1T508D, had a similar level of FRET to that of wild-type LIMK1 (Figure 7A; Figure 7B shows quantification from multiple cells). Thus, the activating phosphorylation of LIMK1T508 is important for the interaction of LIMK1 with fascin-1. To relate these findings to filopodia assembly, the possible co-localization of wild-type or fascin-binding or non-binding mutant forms of GFP-LIMK1 with mRFP-fascin-1 was examined by confocal microscopy. Fascin-1 was located along the length of each linear filopodium, and LIMK1 was detected at the base of filopodia (Figure 7C; see Additional file 6, Figure S3C). Similar observations were made with cells co-expressing mRFP-lifeact and GFP-LIMK1 (see Additional file 6, Figure S3D). These observations are in line with other reports on the cellular localization of LIMK1: typically, LIMK1/2 are mostly cytosolic, without bulk co-localization with the actin cytoskeleton or substrates such as cofilin [39,40]. Cells expressing GFP-LIMKT508A showed fascin-1/LIMK co-localization in some areas of protrusions (Figure 7C), while cells expressing GFP-LIMK1D460A had fewer filopodia that contained less fascin-1 (Figure 7C, D). As quantified from the static images, cells expressing GFP only, GFP-LIMK1, or GFP-LIMK1T508A formed equivalent numbers of filopodia, and in each case the filopodia were around 3 μm in length (Figure 7C,D). In cells expressing GFP-LIMK1D460A, the few filopodia that formed were around 2 μm in length (Figure 7C,D).

**Figure 8:**
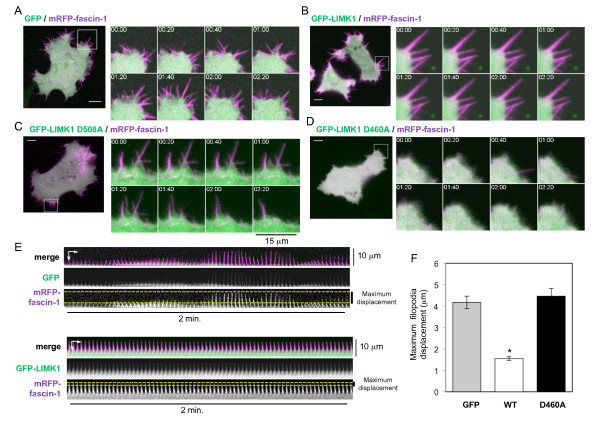
**The fascin-1/p-Lin-11/Isl-1/Mec-3 kinase (LIMK)1 interaction promotes stabilization of filopodia**. **(A-D) **Images from confocal time-lapse movies of cells expressing mRFP-fascin-1 with **(A) **green fluorescent protein (GFP), **(B) **GFP-LIMK1 (B), **(C) **GFP-LIMK1T508A or **(D) **GFP-LIMK1D460A. see Additional files 7 to 10 (movies 4 to 7). There were 15 to 25 cells per condition analyzed in 4 independent experiments. and cells from representative movies are shown. **(A-D) **Boxed 15 × 15 mm regions in the lefthand panels are enlarged in the images from a series of time points in the right panels. See Additional file 3, Figure S3, for single-channel images from **(A) **and **(B)**. Scale bars, 10 μm. **(E) **Kymographs of representative filopodia from cells co-expressing GFP or GFP-LIMK1 and mRFP-fascin-1. Displacement of filopodia was measured in accordance with the maximum change in position of filopodial tips over 2 minutes, as indicated by the dotted yellow lines. **(F) **Quantification of the maximum displacement of filopodia from cells expressing GFP, or wild-type or mutant GFP-LIMK1 and mRFP-fascin-1. Each column represents the mean from five filopodia from at least five cells per condition in four independent experiments; bars indicate SEM. *P < 0.001 versus GFP control.

The requirement for kinase activity of LIMK1/2 in filopodia formation is in line with the known roles of LIMK1/2 in stabilization of F-actin cytoskeleton [36,41,42]. The effects of expression of wild-type or mutant LIMK1 on filopodia dynamics in migratory SW480 cells were therefore examined by time-lapse confocal microscopy. Kymography was also carried out to visualize physical displacement of individual filopodia over time. Compared with cells expressing GFP only, the filopodia of GFP-LIMK1-expressing cells had a longer life (Figure 8A, B), as measured by reduced displacement of the tips of filopodia over time (Figure 8E; Figure 8F shows quantification from multiple cells; see Additional file 7, movie 4; see Additional file 8, movie 5). By contrast, the filopodia of cells expressing GFP-LIMK1T508A, which does not interact with fascin-1, had motility equivalent to the filopodia of control cells (Figure 8C; see Additional file 9, movie 6). Cells expressing catalytically inactive GFP-LIMK1D460A initiated filopodia that collapsed and did not persist, thus leading to fewer and smaller filopodia, in agreement with the static images (Figure 8D; see Additional file 10, movie 7). The motility and displacement over time of the few filopodia that did form in GFP-LIMK1D460A-expressing cells were equivalent to the behavior of filopodia of control cells (Figure 8F, see Additional file 10, movie 7). Thus, promotion of the fascin-1/actin interaction stimulates the stability and persistence of filopodia.

## Discussion

Several lines of indirect evidence have linked the formation of fascin-containing cell protrusions with the status of actomyosin contractility or focal adhesions, but the processes involved, in particular the role of Rho GTPase, have remained obscure. In this study, we established, with multiple lines of evidence, that Rho activity modulates the ability of fascin-1 to interact with actin in both normal and carcinoma-derived cells. The discovery of this novel function of Rho was advanced by the development of a novel assay to measure the fascin-1/actin interaction by FRET/FLIM microscopy. In this assay, a small, actin-binding peptide, lifeact, was adopted as the FRET donor. Lifeact binds reversibly to F-actin, and thus FRET with fascin-1 takes place only when both molecules are in close proximity (<10 nm) and bound to filamentous actin. The specificity of the interaction measured was established by: 1) the lack of FRET interaction of lifeact with the non-actin-bundling form of fascin-1, fascin-1S39D; 2) the independence of fascin-1/lifeact FRET from PKC kinase activity, and 3) the correspondence of lifeact distribution and fascin-1/lifeact FRET with a subset of the F-actin structures to which phalloidin binds.

Using the combined approaches of immunofluorescence microscopy, time-lapse imaging of cells expressing GFP-fascin-1, and the novel FRET assay, we found that inhibition of either Rho or its effectors Rho kinase I and II resulted in inhibition of the fascin-1/lifeact interaction as measured by FRET/FLIM. According to the indirect immunofluorescence of endogenous F-actin or fascin-1 and the imaging of live cells expressing GFP-fascin-1, inhibition of either Rho or Rho kinases also altered cell morphology and led to the formation of more protrusions containing fascin-1 at cell edges. When analyzed in detail by confocal time-lapse microscopy, the filopodial activity of Y27632-treated cells was seen to occur within regions of increased membrane ruffling. These filopodia had distinct characteristics: GFP-fascin-1 was not localized strongly along the filopodial shaft; the filopodia were less straight, probably as a result of loss of actin-bundle rigidity caused by decreased localization of fascin-1 towards filopodial tips (Figure 4), and the lifetimes of these filopodia were longer (Figure 4; see Additional file 3, movie 1; see Additional file 4, movie 2; see Additional file 5, movie 3).

The FRET assay studies a specific molecular interaction within the overall assembly/disassembly dynamics of protrusions. Because Rho kinase inhibition of SW480 migration on LN is fascin-1-independent [5], it is likely that control of protrusion number has a multifactorial basis, for example, it may be linked to both actomyosin-based cell tension and chemical signaling. Inhibition of Rho or Rho kinases is well known to inhibit stress-fiber microfilament bundles within cell bodies [43,44]; however, in our experiments, many cells treated with these inhibitors displayed increased association of fascin-1 with cell-body microfilament bundles. Fascin-1 and tropomyosin act as antagonists for actin binding, and previous studies of ECM-adherent cells have indicated preferential association of fascin-1 with cell-body microfilament bundles in cells under conditions of reduced focal adhesion assembly and contractility [4,20,24]. Treatments with C3 toxin or Y27632 are likely to lead to similar conditions of reduced cell contractility in adherent cells.

By combining the new lifeact FRET assay with biochemical analyses of cell extracts, we have identified Rho-dependent regulation as a novel signaling process that affects both fascin-1 and the fascin-1/actin interaction. Importantly, the mechanistic basis for the pathway downstream of Rho and Rho kinases resides in a previously unidentified process, the interaction of LIMK1/2 with fascin-1. Both of the LIMK isoforms are expressed in many tissues, although specificity of expression patterns and subcellular localizations has been described [37,45,46]. In this study, we identified the interaction of fascin-1 with active LIMK1/2 in SW480 cells by biochemical pull-down from cell extracts and by FRET/FLIM in intact, migrating cells. As established by the immunoblots, fascin-1 pull-down experiments, confocal microscopy, and FRET/FLIM analysis for LIMK1, the mechanism of the interaction depends on LIMK1 activation by Rho kinase phosphorylation, but is independent of the status of S39-phosphorylation of fascin-1. Both S39-phosphorylated and non-phosphorylated fascin-1 are needed for efficient migration of SW480 cells [5], and these new data suggest that the LIMK1/fascin-1 interaction is a possible mechanism to retain S39-phosphorylated fascin-1 in proximity to actin structures at cell edges. We found that fascin-1 localized in filopodia in cells expressing wild-type or T508A mutant forms of LIMK1; however, only wild-type LIMK1 was competent to stabilize filopodia. LIMK1-T508A, which did not interact with fascin-1 in the FRET assay, did not increase filopodial persistence. Thus the LIMK1/fascin-1 interaction contributes to the stability of filopodia. As reported previously [41,42], expression of kinase-dead LIMK1 reduced in decreased numbers of filopodia, and the remaining filopodia were not stabilized.

**Figure 9:**
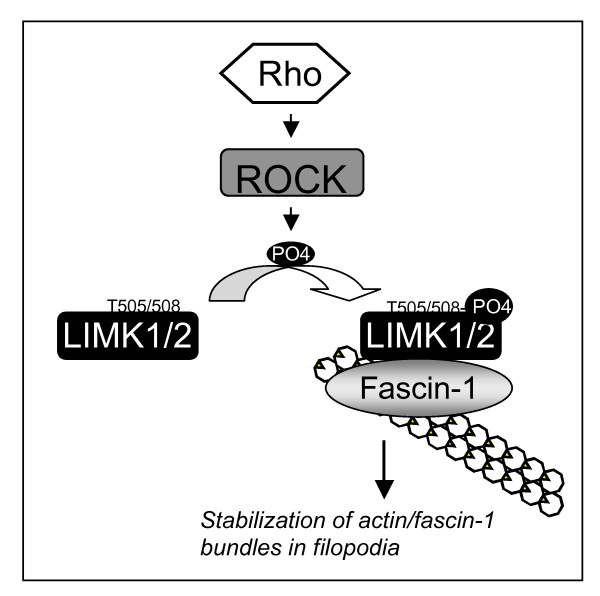
**Model for the novel pathway that regulates fascin-1/actin interaction and filopodia stability**. See Discussion for details. As shown in Figure 6, active p-Lin-11/Isl-1/Mec-3 kinase (LIMK)1/2 bound to both S39-phosphorylated and non-phosphorylated fascin-1.

Overall, these results identify a novel pathway that regulates actin-binding by fascin-1, with functional consequences for filopodial stability (Figure 9). Regulation of the fascin-1/actin interaction did not depend on activity of MLCK or on myosin ATPase activity, and is thus separable from the status of actomyosin contractility.

Similar to fascin-1, LIMK1 and LIMK2 contribute functionally to cancer cell invasion and metastasis, and are therefore of interest as therapeutic targets [36,47,48]. Thus, fascin-1 and LIMK1/2 might co-operate to promote protrusions and cell motility if they are co-expressed in tumors. For example, LIMK1/2 overexpression is well known to increase F-actin, protrusions, and stress fibers in various cells, and correspondingly, LIMK1/2 knockdown or expression of kinase-dead LIMK1/2 decreases F-actin stability, filopodia/lamellipodia, and cell migration [41,42,48]. We propose that the cell protrusion phenotypes observed after LIMK1/2 inhibition may, in part, be mediated by reduced actin binding and bundling by fascin-1. Interestingly, LIMK1/2 are also downstream effectors of p21-activated kinases (PAK), which, as we have previously shown regulate formation of the fascin-1/cPKC complex in colon carcinoma cells, and which also correlate clinically with metastatic progression [19,49]. Thus, LIMK1/2 might represent a nexus between several pathways that regulate the ability of fascin-1 to participate in different protein complexes.

A well-characterized substrate of LIMK1/2 is cofilin, an actin-binding protein that acts to depolymerise F-actin by its actin-severing activity. Phosphorylation of cofilin on ser-3 by LIMK1/2 inhibits its severing activity, resulting in the aforementioned stabilization or reorganization of actin structures [50-52]. Similar to fascin-1, the cofilin pathway has been linked to breast-cancer metastasis [50]. It was recently shown that cofilin associates transiently with retracting filopodia, and that its actin-severing activity is enhanced on actin bundles crosslinked by fascin-1 [53]. The relationship between the fascin-1/LIMK1/2 complex and cofilin in the dynamics of filopodia are questions of future interest. It will be important to establish if fascin-1 competes with cofilin for LIMK1 kinase availability, thereby modulating actin dynamics directly, or whether LIMK1/2 act directly or indirectly as a scaffold to maintain fascin-1 in proximity to F-actin. Alternatively, fascin-1 binding might inhibit the kinase activity of LIMK1/2, thereby affecting actin dynamics indirectly, as previously shown for nischarin [54]. LIMK1 contributes to the transcriptional activity of serum response factor [55], and it will be interesting to determine if this activity is modulated by fascin-1.

## Conclusions

In this study, we have identified a novel activity of the small GTPase Rho in promoting the interaction of fascin-1 with actin in ECM-adherent, migratory cells. Rho, Rho kinases, and LIMK1/2 were identified as key effectors, and activated LIMK1 or LIMK2 act as novel binding partners of fascin-1. These findings have many implications for our understanding of how fascin-1 protein complexes and actin dynamics are coordinated in contractile and protrusive actin-based structures in intact cells, and may be particularly relevant to carcinoma cell migration and metastasis. In the course of the study, we developed a novel method for analysis of the fascin-1/actin interaction by FRET/FLIM. This method has potential general applicability for analyzing the activities of actin-binding proteins in intact cells.

## Methods

### Cell lines and materials

C2C12 mouse skeletal myoblast and SW480 human colon carcinoma cells were from the American Type Culture Collection (ATCC). GFP-fascin-1 and PKCγ-mRFP constructs were prepared as previously described [15,17]. PKCα-mRFP [56] was a gift from T. Ng, and the GFP-tagged forms of LIMK1, LIMK2 [57], ROCKI, and ROCKII were gifts from G. Jones (both King's College London, London, UK). GFP-caldesmon and the deletion mutant GFP-caldesmon-445 [35] were a gift from Alexander Bershadsky (Weizman Institute of Science). The Ras-binding domain-glutathione S-transferase (RBD-GST) plasmid was the gift of M. Schwartz (Yale University) [58]. GFP-lifeact [32] was a gift from R. Wedlich-Soldner (MPI, Munich, Germany).

The mRFP-fascin-1S39A and mRFP-fascin-1S39D constructs were prepared by subcloning cDNAs for human fascin-1S39A and fascin-1S39D into pcDNA3-mRFP(N) using the PCR primers shown in Table 1. mRFP-fascin-1 cDNA was generated from GFP-fascin-1WT by subcloning into pCR-Blunt (Invitrogen Corp., Carlsbad, CA, USA), using the PCR primers shown in Table 1, and then subcloned into pcDNA3-mRFP(N) using *Hin*dIII and *Ec*oRI restriction sites. A vector encoding GFP-fascin-1 under control of a truncated CMV promoter (specGFP-fascin-1) was generated by replacing the CMV promoter by the speckled promoter from a GFP-paxillin plasmid [59] (gift of R. Horwitz, University of Virginia) using *Ase*I and *Nhe*I restriction sites. GFP-LIMK1/2 mutant constructs were generated by site-directed mutagenesis using the oligonucleotide pairs shown in Table 1.

**Table 1:** Primer and oligonucleotide pairs used for PCR, cloning and site-directed mutagenesis

Name	Direction	Sequence
fascin-1S39A/D cDNAs	Forward	GTACGGATCCATGACCGCCAACGGCACAGCC
Subclonings into pcDNA3.mRFP(N)	Reverse	GTACGAATTCCTAGTACTCCCAGAGCGAGGC

fascin-1 cDNA	Forward	CCGGGAAGCTTCGCAGCGGCCTCTCG
Subcloning into pCR-Blunt	Reverse	GACGGGCCTAGGCTAGTACTCCCAGAG

LIMK1/460A mutagenesis	Forward	GAACATCATCCACCGAG**C**CCTCAACTCCCACAAC

	Reverse	GTTGTGGGAGTTGAGG**G**CTCGGTGGATGATGTTC

LIMK1/508A mutagenesis	Forward	GCAAGAAGCGCTAC**G**CCGTGGTGGGCAAC

	Reverse	GTTGCCCACCACGG**C**GTAGCGCTTCTTGC

LIMK2/451A mutagenesis	Forward	GTGCATCATCCACCGGG**C**TCTGAACTCGCACAACTG

	Reverse	CAGTTGTGCGAGTTCAGA**G**CCCGGTGGATGATGCAC

LIMK2/505A mutagenesis	Forward	CGCAAGAAGCGCTAC**G**CGGTGGTGGGAAACC

	Reverse	GGTTTCCCACCACCG**C**GTAGCGCTTCTTGCG

For bacterial expression of 6His-tagged fascin-1, cDNAs encoding wild-type, S39A, or S39D human fascin-1 from the pEGFP-fascin-1 plasmids were cloned between the *Not*I and *Xho*I restriction sites of pET-30a(+) (Novagen). The reading frame was adjusted by subsequent digestion and blunting of the *Not*I site. BIM, Y27632, ML7, BDM and blebbistatin were obtained from Calbiochem (San Diego, CA, USA). C3 endotoxin and RhoA G-LISA kit were from Cytoskeleton Inc (). Antibodies used included mouse monoclonal antibody to fascin-1 (clone 55k-2; Dako, Glostrop, Denmark); to LIMK1, LIMK2 and phosphoLIMK1/2 (Cell Signaling Technology, Beverly, MA, USA) and to Rho, ROCK I and II (BD Transduction Labs).

### Cell-extracellular matrix adhesion and Immunofluorescence

C2C12 cells were maintained in DMEM containing 20% fetal calf serum (FCS), and SW480 cells were maintained in DMEM with 10% FCS. For transient transfections, cells were plated at 30% to 40% confluency, and transfected with transfection reagents (Polyfect (Qiagen Inc., Valencia, CA, USA or Lipofectamine 2000 (Invitrogen Corp.)) in accordance with the manufacturer's instructions. At 48 hours post-transfection, cells were re-plated as single cell suspensions onto glass surfaces coated with 50 nmol/L FN (Calbiochem) or Engelbreth-Holm-Swarm (EHS) laminin (Sigma-Aldrich, St Louis, MO, USA) under serum-free conditions, as described previously [5,18]. In experiments involving pharmacological inhibitors, cells were pretreated with the agent for 30 minutes prior to the adhesion assays being set up, and the agent was maintained in the medium throughout the assay. The concentrations of inhibitors to be used were established in pilot experiments, and the lowest concentrations that affected cell morphology and actin organization reproducibly without evidence of cytotoxicity, as determined by Trypan blue exclusion, were chosen for the experiments. After 1 (C2C12) or 2 (SW480) hours at 37°C to allow adhesion and initiation of random cell migration, non-adherent cells were removed by rinsing in Tris-buffered saline (TBS) and adherent cells were fixed in 2% paraformaldehyde (EMS) and permeabilized in TBS with 0.5% Triton-X100 for staining with tetramethyl rhodamine isothiocyanate-phalloidin (Sigma), phalloidin-633, or antibodies to vinculin (clone h-VIN1; Sigma) or phosphotyrosine (clone 4G10; Millipore Corp., Billerica, MA, USA). For fascin-1 staining, cells were fixed in absolute methanol, and for LIMK1/2 staining, they were fixed in 4% paraformaldehyde. Digital images were taken at room temperature under the ×63 objective of a laser scanning confocal microscope using confocal acquisition software (Leica, Heerbrugg, Switzerland). Morphometric analysis of adherent C2C12 cells was carried out by measuring cell areas and the numbers and lengths of individual peripheral fascin or F-actin bundles from calibrated digital images using the software Improvision Openlab (Perkin Elmer, Waltham, MA, USA).

### Fluorescence resonance energy transfer/fluorescence lifetime imaging microscopy

FLIM was performed cells transfected with specified constructs, with fixation and data analyzed as described previously [60]. Details of time-domain FLIM performed with a multiphoton microscope system have been described previously [60]. FLIM capability was provided by time-correlated single-photon-counting electronics (SPC 700; Becker & Hickl, Berlin, Germany). Widefield acceptor (mRFP) images were acquired using a charge-coupled device camera (Hamamatsu Photonics, Hamamatsu, Japan) at exposure times of <100 ms. Data were analyzed using TRI2 software (developed by Dr P. Barber). All histogram data were plotted as mean FRET efficiency from >10 cells per sample. Lifetime images of exemplary cells are presented using a pseudocolor scale, with blue depicting normal GFP lifetime (that is, no FRET) and red depicting reduced GFP lifetime (areas of FRET). Each experiment was repeated at least three times.

### Live cell imaging

SW480 cells were transfected with GFP-fascin-1, and 24 hours later, were plated onto glass-bottomed imaging chambers (LabTek; Nunc) coated with 15 nmol/l laminin for 6 hours before treatment with DMSO (solvent control) or 10 μmol/l Y27632 for 30 minutes. Live confocal microscopy imaging was performed on a microscope (A1R; Nikon, Tokyo, Japan) equipped with a 40× oil objective lens (CFI Plan Fluor; Nikon) at 37°C. Images were captured and exported with NIS Elements software (Nikon). Movies were acquired as one image per 2 seconds over 60 to 150 frames, and are presented at playback rates of 15 frames/second. Morphometric analyses of filopodia formation in SW480 cells were performed by measuring the number and length of GFP-tagged or RFP-tagged fascin-positive filopodia, and by line-scan analysis of fluorescence intensity along the length of single, randomly selected filopodia. To analyze the dynamics of an individual filopodium, kymographs of 2 × 10 μm areas encompassing one filopodium were selected, and the montage was performed in the X-axis. The mRFP-fascin-1 signal was thresholded, the front of the filopodium outlined, and the resulting coordinates used to calculate several dynamic values including maximum filopodia displacement. All image analyses were performed with ImageJ software (http://rsb.info.nih.gov/ij/download.html). Each experimental condition was tested in at least three independent experiments.

### Immunoblotting and fascin-1 pull-downs

Adherent cells for immunoblotting were extracted on ice in 1% Triton X-100 in TBS containing protease inhibitor cocktail (Roche Diagnostics, Basel, Switzerland) for 12 minutes. SDS-PAGE and immunoblotting with selected antibodies and enhanced chemiluminescence (ECL) detection was carried out as described previously [5]. Recombinant 6His-tagged fascin-1 was expressed in BL21 *E. coli *by induction with 1 mmol/l isopropyl β-D-1-hiogalactopyranoside (IPTG; Sigma-Aldrich) for 5 hours at 25°C. Bacterial pellets were resuspended in buffer(300 mmol/l NaCl, 10 mmol/l imidazole and 50 mmol/l Na_2_HPO_4 _pH 8), then sonicated and clarified. Lysates were passed over nickel-nitrilotriacetic acid (Ni-NTA) beads (Qiagen Inc.) to allow fascin-1 binding, beads were washed (300 mmol/l NaCl, 25 mmol/l imidazole and 50 mmol/l Na_2_HPO_4 _pH 8), and fascin-1 was eluted (300 mmol/l NaCl, 250 mmol/l imidazole in 50 mmol/l Na_2_HPO_4 _(pH 8), and dialyzed against buffer (150 mmol/l NaCl, 10 mmol/l imidazole and 50 mmol/l Na_2_HPO_4_, pH 8). For pull-down experiments, the wild-type or mutant fascin-1 proteins were immobilized on Ni-NTA beads in disposable polystyrene chromatography columns (Thermo Fisher Scientific Inc., Rockford, IL, USA ). SW480 cell lysates were extracted (1% Triton, 150 mmol/l NaCl, 2 mmol/l MgCl_2 _and 10 mmol/l imidazole pH 7.5), then passed through the columns, washed extensively (1% Triton, 150 mmol/l NaCl, 2 mmol/l MgCl_2 _and 25 mmol/l imidazole, pH 7.5), and the bound proteins were eluted (1% Triton, 150 mmol/l NaCl, 2 mmol/l MgCl_2 _and 250 mmol/l imidazole pH 7.5). The eluted proteins were mixed with SDS-sample buffer and subjected to SDS-PAGE and immunoblotting as described previously [5].

### Rho activity assays

Rho-GTP pull-down assays were carried out on extracts from C2C12 cells adherent on 30 nmol/l FN for 1 hour with a GST fusion protein containing the effector domain of rhotekin (RBD-GST) as described previously [58]. All extracts contained equivalent total Rho protein, as measured by immunoblotting with anti-Rho (Transduction Labs) and ECL detection. Rho-GTP levels were compared with Rho status in C2C12 cells adherent on 30 mmol/l thrombospondin-1 for 1 hour or suspended for 90 minutess over BSA-blocked dishes. RhoA-GTP levels were also quantified with a G-LISA kit used in accordance with manufacturer's instructions (Cytoskeleton Inc). Lysates were prepared from SW480 cells plated on 15 nmol/l laminin for 2 hours, either after 4 hours of treatment with DMSO (control) or 1 μmol/l C3, or 30 minutes of pre-treatment with 1 μmol/l BIM.

### Statistical analysis

Data were analyzed by descriptive statistics. *P *values were calculated using a two-tailed Student's *t*-test for unpaired samples Triplicate samples were scored in each experiment, and three to four independent experiments were carried out for each experimental condition. ANOVA was used to test statistical significance between different populations of data. Statistical analysis was performed with normalized data by one-way ANOVA and subsequent multiple range test between conditions. *P *< 0.05 was considered significant.

## List of abbreviations

BDM: 2,3-Butanedione monoxime; BIM: Bisindolylmaleimide I; BSA: Bovine serum albumin; DMSO: Dimethyl sulfoxide; FN: fibronectin; FRET/FLIM: fluorescence resonance energy transfer/fluorescence lifetime imaging microscopy; GFP: green fluorescent protein; LIMK: p-Lin-11/Isl-1/Mec-3 (LIM) kinase; LN: laminin; MLCK: myosin light chain kinase; mRFP: monomeric red fluorescent protein; PKC: protein kinase C; Rho kinase: Rho-associated coiled-coil-forming kinase; TBS: Tris-buffered saline.

## Competing interests

The authors declare that they have no competing interests.

## Authors' contributions

JCA began the initial experiments. JCA and MP devised the study. AJ, MP, and JCA carried out the experiments. JCA wrote the manuscript with input from MP and AJ. All authors approved the final version.

## Supplementary Material

Additional file 1 **Figure S1**. **(A) **Demonstration that SW480 cells migrating on laminin (LN) contain active Rho A, and that Rho is effectively inhibited by C3 exotoxin under the experimental conditions. **(B,C) **Co-localization of GFP-lifeact signal with phalloidin-633 signal and mRFP-fascin-1 in areas of high fluorescence resonance energy transfer (FRET) efficiency in SW480 cells on LN transiently expressing GFP-lifeact and monomeric red fluorescent protein (mRFP)-fascin-1. Boxed area in **(B) **is shown enlarged for each single-channel image and the lifetime image in **(C)**. Scale bars, **(B) **10 μm; **(C) **5 μm. Click here for file

Additional file 2 **Figure S2**. **(A) **Immunoblots for expression of Rho kinase isoforms, with 25 μg of cell extract was loaded per lane. Molecular mass markers are in kDa. **(B) **Measurement of the interaction of monomeric red fluorescent protein (mRFP)P-fascin-1S39A with GFP-lifeact in C2C12 and SW480 cells, without or with Rho kinase inhibition. Cells transiently transfected with the indicated plasmids were plated on laminin (LN) for 2 hours, without or with pre-treatment with Y27632, then fixed, mounted, and imaged using fluorescence lifetime imaging microscopy (FLIM) to measure fluorescence resonance energy transfer (FRET). In each panel, intensity multiphoton GFP (donor) images are shown with the corresponding epifluorescence image for mRFP (acceptor). Lifetime images are presented in a blue-to-red pseudocolor scale with red as short lifetime. **(C) **Reduced filopodial localization of fascin-1 upon inhibition of Rho kinases. Single -hannel images of filopodia are shown from the same cells as presented in the merged images in Figure 4D. Scale bars, 10 μm. Click here for file

Additional file 3 **Time-lapse confocal image avi movie 1**. Monomeric red fluorescent protein (mRFP)-lifeact dynamics in the same SW480 cell before and after treatment with Y27632. Boxed 25 × 25 μm squares in the lefthand panel are zoomed in on the righthand panel. Time-lapse series were recorded every 2 seconds for 2 minutes, per cell and treatment. Click here for file

Additional file 4 **Time-lapse confocal image avi movie 2**. A representative SW480 cell transiently expressing green fluorescent protein (GFP)-fascin-1 and monomeric red fluorescent protein (mRFP)-lifeact. Zoomed images represent the 25 × 25 μm box from the top-left panel. Images were acquired every 2 sec over 4 minutes. Click here for file

Additional file 5 **Time-lapse confocal image avi movie 3**. A representative SW480 cell transiently expressing green fluorescent protein (GFP)-fascin-1 and monomeric red fluorescent protein (mRFP)-lifeact after treatment with Y27632. Zoomed images represent the 25 × 25 μm box from the top-left panel. Images were acquired every 2 sec over 4 minutes. Click here for file

Additional file 6 **Figure S3**. **(A) **Co-localization of fascin-1 and LIMK1/2 in SW480 cells on laminin (LN). Confocal images of SW480 cell transiently expressing low levels of green fluorescent protein (GFP)-fascin-1 (expressed under a truncated CMV promoter 'SpecGFP'; see Methods), were fixed and stained for endogenous p-Lin-11/Isl-1/Mec-3 kinase (LIMK)1 (LH panel) or LIMK2 (RH panel). Arrows show examples of concentrations of LIMK1 or LIMK2 that colocalized with fascin-1 at cell peripheries. Scale bars, 10 μm. **(B) **Demonstration that LIMK1/2 activity but not total LIMK1 protein is decreased in cells after treatment with Rho inhibitor (C3 exotoxin) or Rho kinase inhibitor (Y27632). Molecular markers are in kDa. Representative of three independent experiments. **(C) **Representative single-channel images of localizations of GFP or GFP-LIMK1 and mRFP-fascin-1 in SW480 cells on LN. Fascin-1 localizes along the shafts of filopodia and GFP-LIMK1 localizes weakly towards the base of filopodia. Right panels show zoomed single-channel and merged images of the boxed 15 × 15 μm regions in the lefthand panels. Scale bars, 10 μm. These images correspond to one timepoint of the merged images in Figure 8A,B. **(D) **Representative confocal images of localizations of GFP-LIMK1 and mRFP-lifeact in SW480 cells on LN. Scale bars, 10 μm. Click here for file

Additional file 7 **Time-lapse confocal image avi movie 4**. A representative SW480 cell expressing green fluorescent protein (GFP) and monomeric red fluorescent protein (mRFP)-fascin-1. Zoomed images represent the 15 × 15 μm square in the lefthand panel. Images were acquired every 2 seconds over 2 minutes. Click here for file

Additional file 8 **Time-lapse confocal image avi movie 5**. A representative SW480 cell expressing green fluorescent protein (GFP)-LIMK1 and monomeric red fluorescent protein (mRFP)-fascin-1. Zoomed images represent the 15 × 15 μm square on the lefthand panel. Images were acquired every 2 seconds over 2 minutes. Click here for file

Additional file 9 **Time-lapse confocal image avi movie 6**. A representative SW480 cell expressing green fluorescent protein (GFP)-LIMK1-T508A and monomeric red fluorescent protein (mRFP)-fascin-1. Zoomed images represent the 15 × 15 μm square on the lefthand panel. Images were acquired every 2 seconds over 2 minutes. Click here for file

Additional file 10 **Time-lapse confocal image avi movie 7**. A representative SW480 cell expressing green fluorescent protein (GFP)-p-Lin-11/Isl-1/Mec-3 kinase (LIMK)1-D460A and monomeric red fluorescent protein (mRFP)-fascin-1. Zoomed images represent the 15 × 15 μm square on the lefthand panel. Images were acquired every 2 seconds over 2 minutes. Click here for file
