# Supplementary figures and images for: A novel Rho-dependent pathway that drives interaction of fascin-1 with p-Lin-11/Isl-1/Mec-3 kinase (LIMK) 1/2 to promote fascin-1/actin binding and filopodia stability
Source: BMC Biol. 2012 Aug 10;10:72. doi: 10.1186/1741-7007-10-72 (PMC3488970; doi:10.1186/1741-7007-10-72)

A

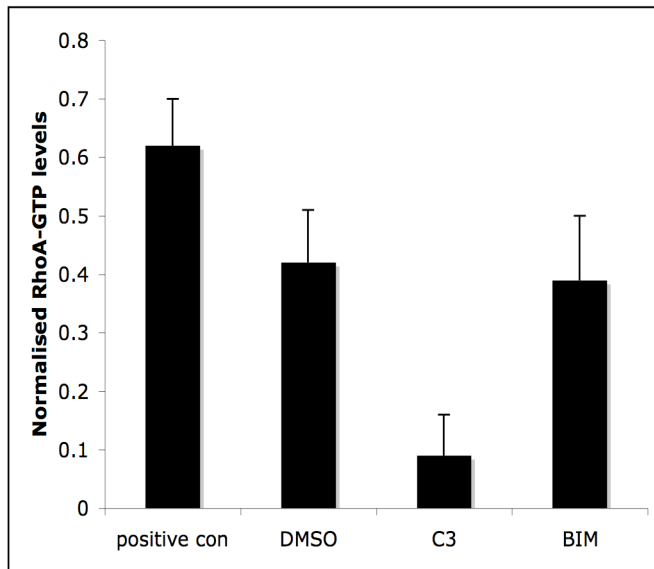

B

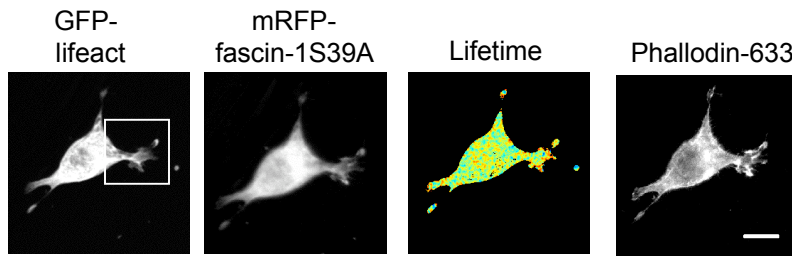

C

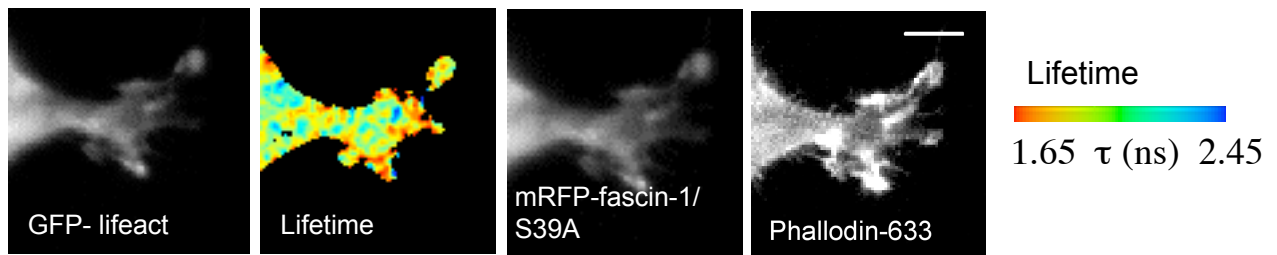

Supplement: Additional file 1 — Figure S1. (A) Demonstration that SW480 cells migrating on laminin (LN) contain active Rho A, and that Rho is effectively inhibited by C3 exotoxin under the experimental conditions. (B,C) Co-localization of GFP-lifeact signal with phalloidin-633 signal and mRFP-fascin-1 in areas of high fluorescence resonance energy transfer (FRET) efficiency in SW480 cells on LN transiently expressing GFP-lifeact and monomeric red fluorescent protein (mRFP)-fascin-1. Boxed area in (B) is shown enlarged for each single-channel image and the lifetime image in (C). Scale bars, (B) 10 μm; (C) 5 μm. [file 1741-7007-10-72-S1.pdf]

Additional item 2 - Fig. S2

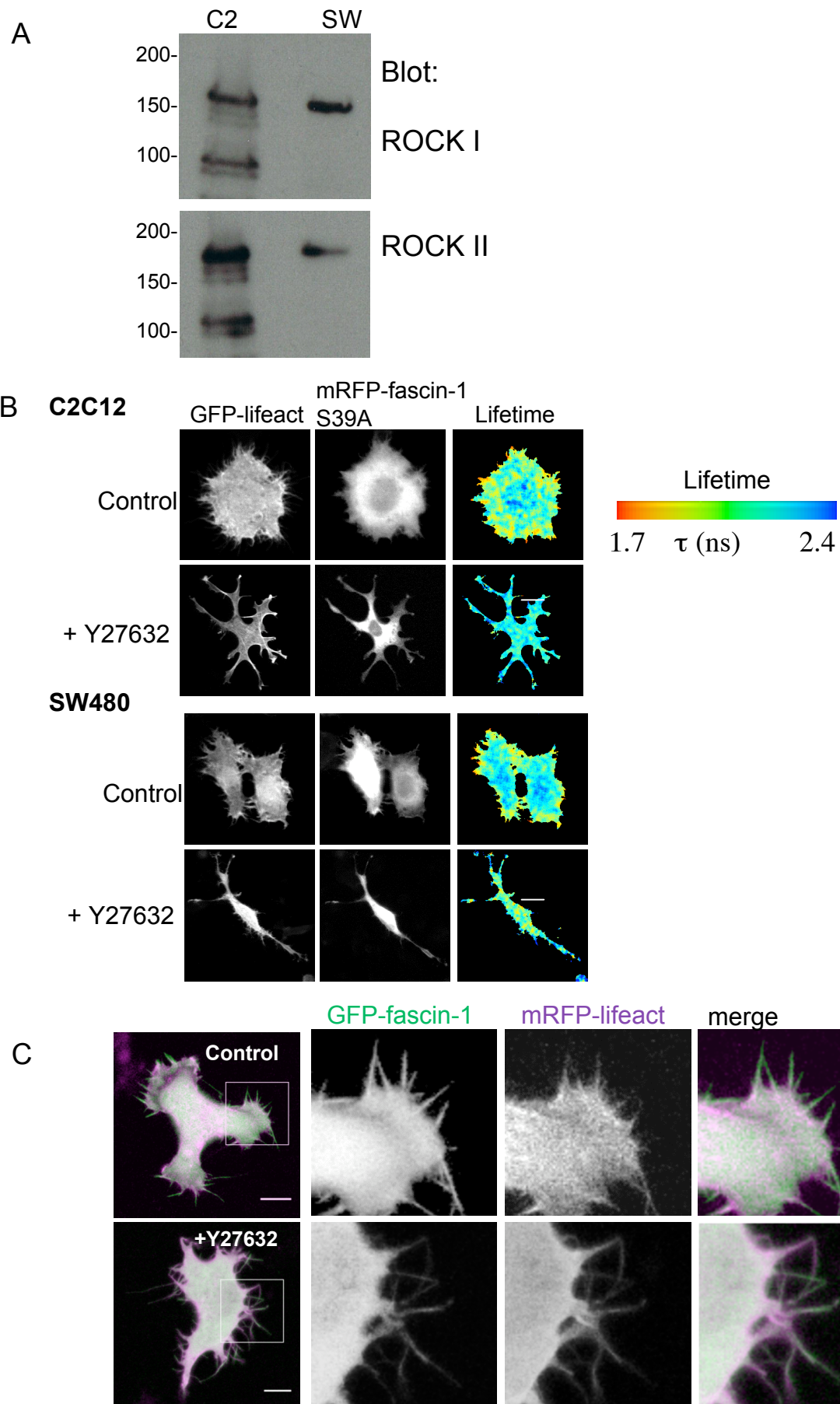

Supplement: Additional file 2 — Figure S2. (A) Immunoblots for expression of Rho kinase isoforms, with 25 μg of cell extract was loaded per lane. Molecular mass markers are in kDa. (B) Measurement of the interaction of monomeric red fluorescent protein (mRFP)P-fascin-1S39A with GFP-lifeact in C2C12 and SW480 cells, without or with Rho kinase inhibition. Cells transiently transfected with the indicated plasmids were plated on laminin (LN) for 2 hours, without or with pre-treatment with Y27632, then fixed, mounted, and imaged using fluorescence lifetime imaging microscopy (FLIM) to measure fluorescence resonance energy transfer (FRET). In each panel, intensity multiphoton GFP (donor) images are shown with the corresponding epifluorescence image for mRFP (acceptor). Lifetime images are presented in a blue-to-red pseudocolor scale with red as short lifetime. (C) Reduced filopodial localization of fascin-1 upon inhibition of Rho kinases. Single -hannel images of filopodia are shown from the same cells as presented in the merged images in Figure 4D. Scale bars, 10 μm. [file 1741-7007-10-72-S2.PDF]

Additional item 3 - Fig. S3

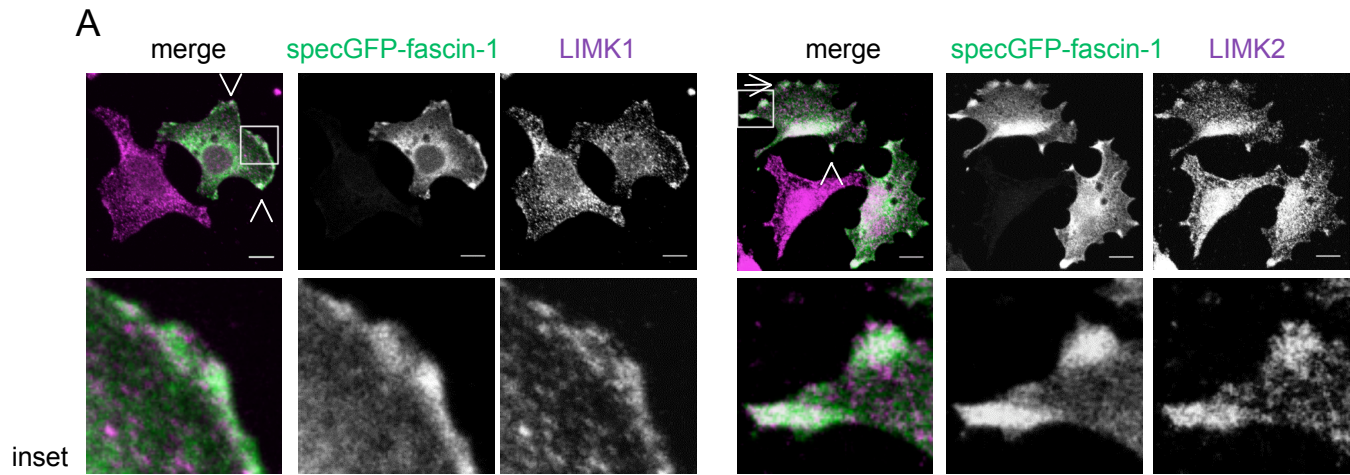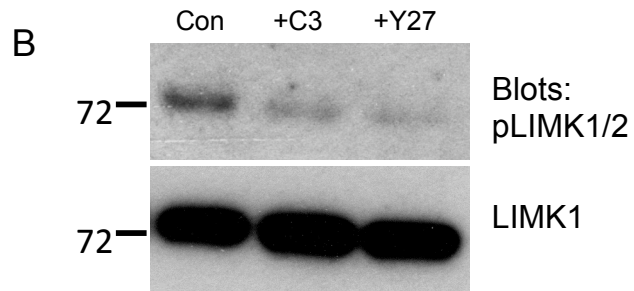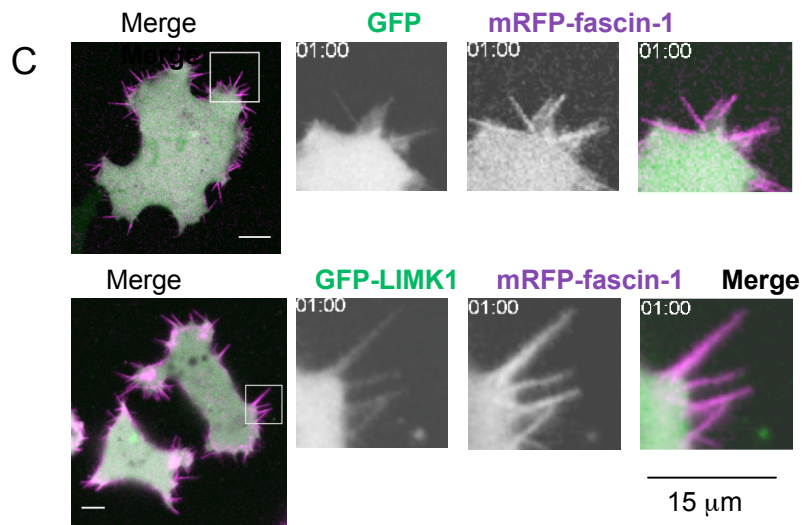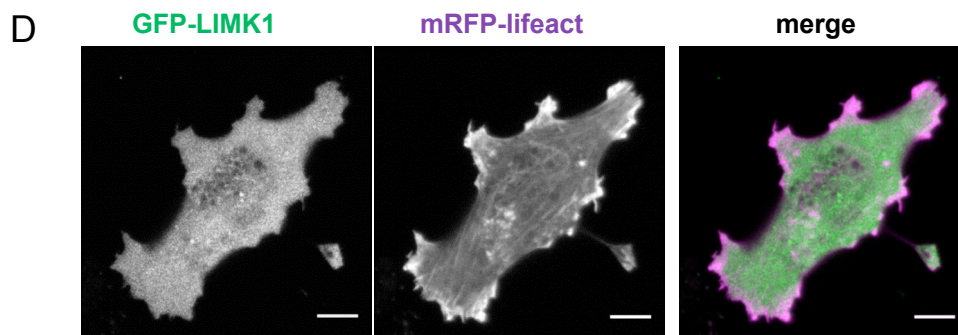

Supplement: Additional file 6 — Figure S3. (A) Co-localization of fascin-1 and LIMK1/2 in SW480 cells on laminin (LN). Confocal images of SW480 cell transiently expressing low levels of green fluorescent protein (GFP)-fascin-1 (expressed under a truncated CMV promoter 'SpecGFP'; see Methods), were fixed and stained for endogenous p-Lin-11/Isl-1/Mec-3 kinase (LIMK)1 (LH panel) or LIMK2 (RH panel). Arrows show examples of concentrations of LIMK1 or LIMK2 that colocalized with fascin-1 at cell peripheries. Scale bars, 10 μm. (B) Demonstration that LIMK1/2 activity but not total LIMK1 protein is decreased in cells after treatment with Rho inhibitor (C3 exotoxin) or Rho kinase inhibitor (Y27632). Molecular markers are in kDa. Representative of three independent experiments. (C) Representative single-channel images of localizations of GFP or GFP-LIMK1 and mRFP-fascin-1 in SW480 cells on LN. Fascin-1 localizes along the shafts of filopodia and GFP-LIMK1 localizes weakly towards the base of filopodia. Right panels show zoomed single-channel and merged images of the boxed 15 × 15 μm regions in the lefthand panels. Scale bars, 10 μm. These images correspond to one timepoint of the merged images in Figure 8A,B. (D) Representative confocal images of localizations of GFP-LIMK1 and mRFP-lifeact in SW480 cells on LN. Scale bars, 10 μm. [file 1741-7007-10-72-S6.PDF]
